# Supplementary material for: In-plane charged antiphase boundary and 180° domain wall in a ferroelectric film
Source: Nat Commun. 2023 Dec 9;14:8174. doi: 10.1038/s41467-023-44091-4 (PMC10710403; doi:10.1038/s41467-023-44091-4)
Supplement: Supplementary file 1 — Supplementary Information [file 41467_2023_44091_MOESM1_ESM.pdf]

Supplementary Information for

Title: In-plane charged antiphase boundary and 180° domain wall in a ferroelectric film

Contents:

Figure S1: X-ray diffraction (XRD) characterization of BFO/LAO films before and after the He-ion implantation.

Figure S2: AFM and PFM characterization of BFO/LAO films before and after the He-ion implantation.

Figure S3: A typical cross-sectional membrane from BFO/LAO films.

Figure S4: Cross-sectional microscopic analysis of the BFO/LAO interface.

Figure S5: RSM results of the He-implanted BFO film.

Figure S6: Simulation of He-ion implantation depth profiles in BFO under different beam energies.

Figure S7: Switching behavior after He-ion implantation.

Figure S8: Reproducibility of IP-CAPBs.

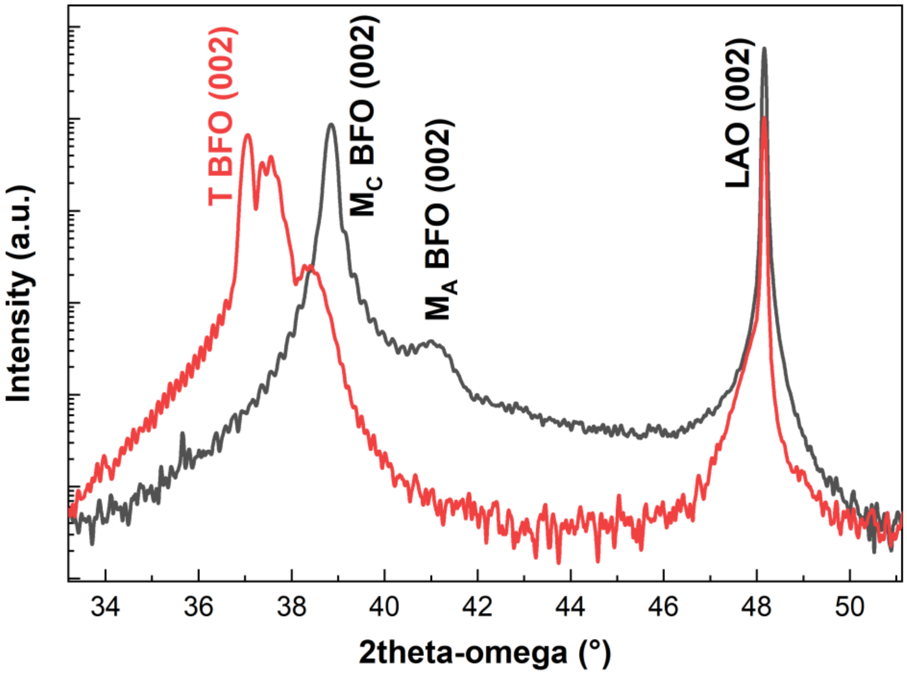

Figure S1: X-ray diffraction (XRD) characterization of BFO/LAO films before and after the He-ion implantation.

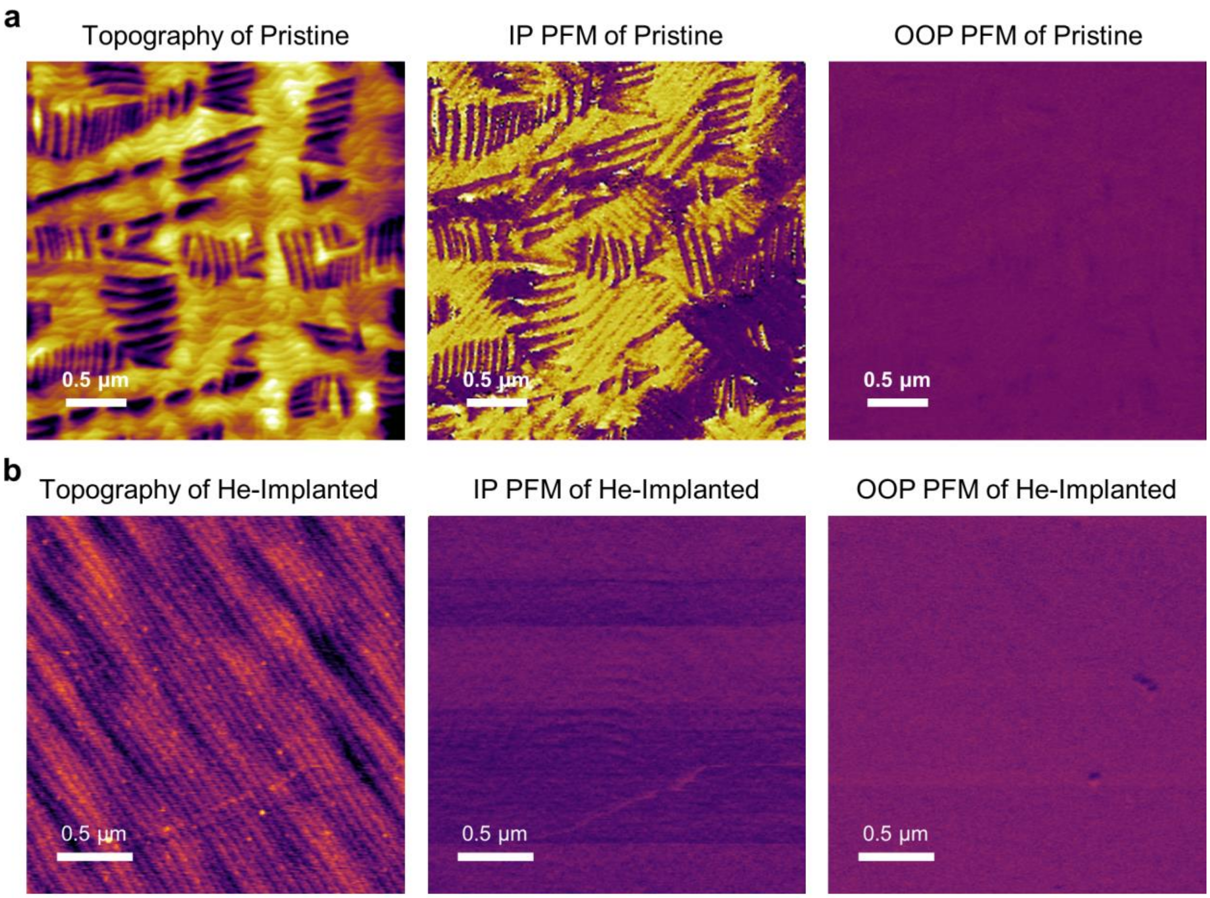

Figure S2: AFM and PFM characterization of BFO/LAO films (a) before and (b) after the He-ion implantation. The same type of maps in (a) and (b) were plotted in the same color scale for clearer comparison. Out-of-plane (OOP) PFM measurements of the same area in BFO/LAO films before and after the He-ion implantation suggests little change in the OOP component of BFO surface polarization, which is consistent with the atomic cross-sectional observation of downward polarization near the surface of both samples.

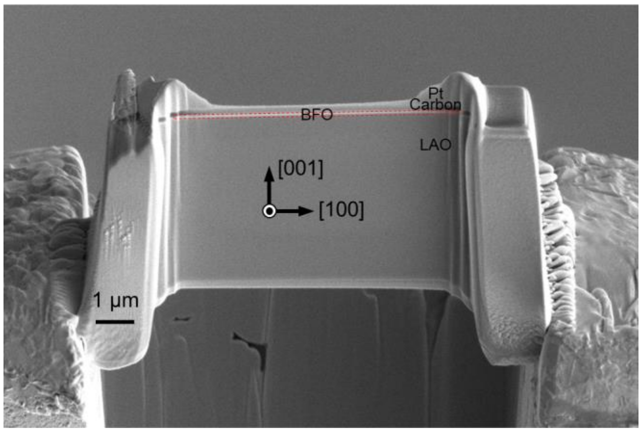

Figure S3: Scanning electron microscopy (SEM) image of a typical cross-sectional membrane from BFO/LAO films for the atomic-resolution characterization. The BFO membrane is cut out along its [100] direction and the membrane is viewed along its [010] zone axis. The BFO is capped by carbon paint and deposited Pt protection layer to prevent surface damage during the Ga-ion milling.

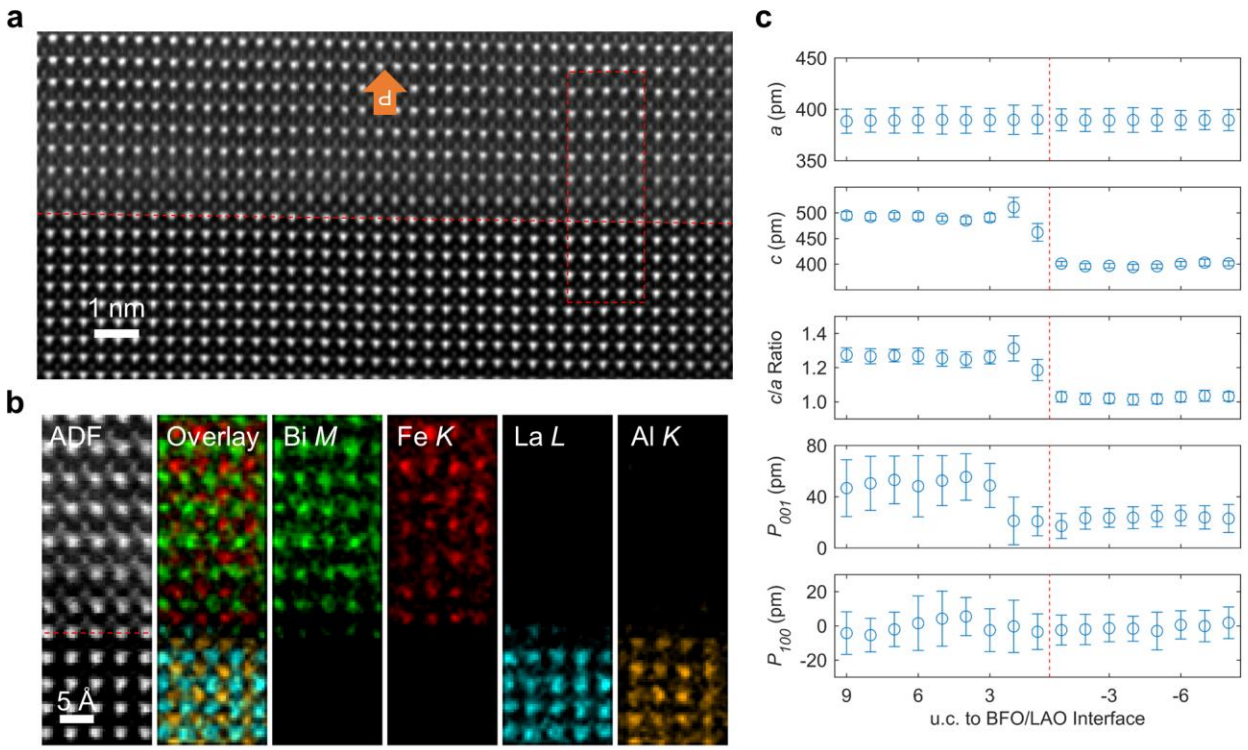

Figure S4: (a) Atomic-resolution HAADF image of the implanted BFO/LAO interface. The upward polarization of BFO near the substrate can be identified from the downward displacement of Fe atomic columns. The elemental analysis of the red dashed rectangle region by EDS is shown in (b) with the simultaneous ADF image, overlaid composition map and the constituent sub-maps presented from left to right. (c) Statistical analyses of the characteristic lattice parameters, averaged from each u.c. row, across the interface from the top (positive u.c. numbers) to the bottom (negative u.c. numbers). The error bars represent the standard deviation of corresponding data from each u.c. row. The interface positions are marked by red dashed lines in every panel.

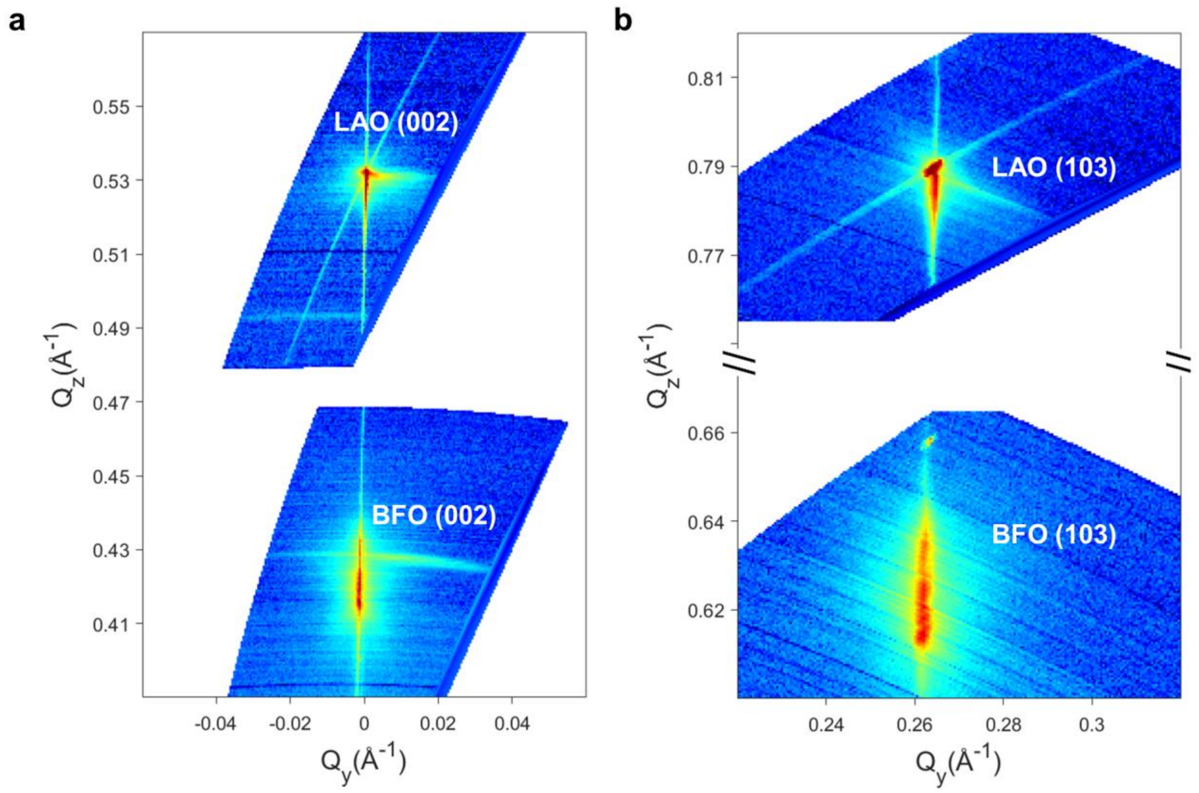

Figure S5: RSM results of the He-implanted BFO film obtained around (a) LAO (002) and (b) (103) spots. We can see that BFO (002) and (103) spots exhibit an elongated shape in contrast to the M_C-like splitting in the pristine BFO (ref. 38: Advanced Functional Materials 2011, 21, 133-138). Furthermore, LAO (103) and BFO (103) spots stay at the same Qy position, demonstrating the strict maintenance of tetragonal epitaxy relation. These results are consistent with the previous report on the true tetragonal transition by He-ion implantation (ref. 35 of the manuscript), proving the symmetry-reduced true tetragonal structure in the He-implanted BFO. The multiple BFO (002) peaks in Figure S1 and the elongated shape of BFO (002) and (103) here may be caused by the varied He-ion densities along the implantation depth direction, leading to a wide distribution of tetragonalities in implanted samples.

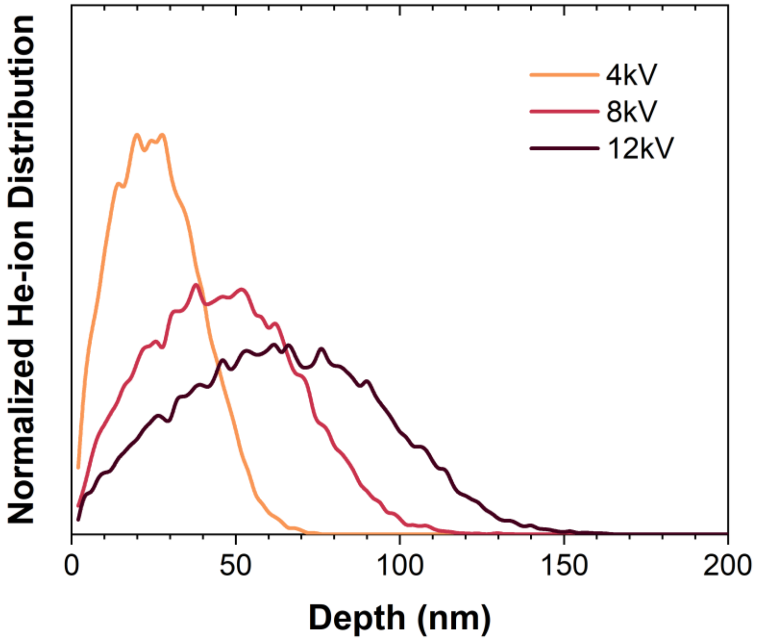

Figure S6: Simulation of He-ion implantation depth profiles in BFO under different beam energies by the open-source software SRIM-2013 (URL: http://www.srim.org/SRIM/SRIMLEGL.htm). Although the penetration depth of 8 kV He can be as deep as 100 nm, the highest He density only reaches a depth of around 60 nm (the depth where we observed the IP-CAPB under 8 kV beam energy) and then the He density drops very fast, which is consistent with our proposed mechanism how He implantation generates the IP-CAPB. It was observed that the oxygen-vacancy density in the top side of IP-CAPBs is higher than that in the bottom side. An antiphase domain is actually a region of anti-site defects in the parent lattice. The switch of A- and B-site ions, leading to anti-site defects, can be caused by the high-energy ion bombardment and assisted by the generated oxygen vacancies. The accumulation and migration of oxygen vacancies and anti-site defects finally compose the IP-CAPB at the implanted depth after the implantation process. Therefore, varied beam energies can lead to different implantation depths and profiles. A different CAPB position can be expected if a different energy is used for the implantation. Since the simulation does not involve the effects caused by the LAO substrate, there may be some slight deviation in the practical depth profiles.

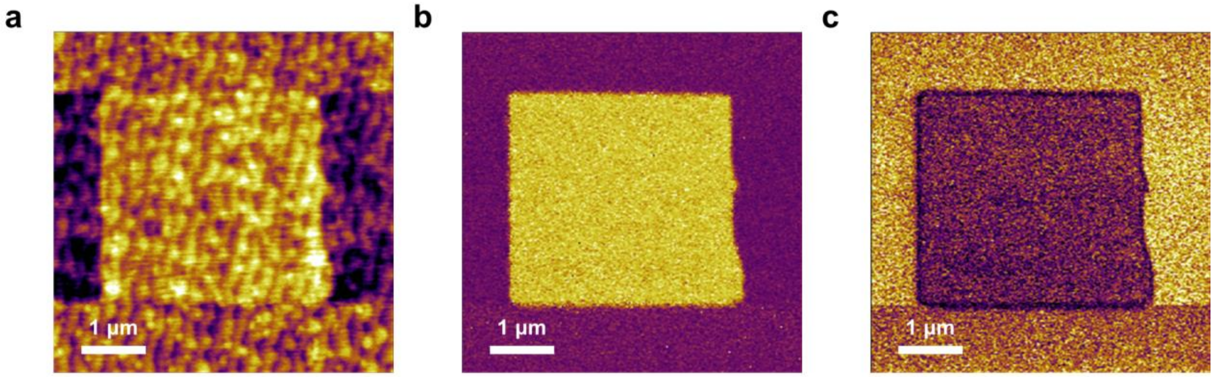

Figure S7: Switching behavior after He-ion implantation. (a) AFM image, (b) out-of-plane PFM phase and (c) amplitude images of a written rectangle in the implanted BFO film. The film was implanted in the same way as reported in the main text with a $Ca_{0.96}Ce_{0.04}MnO_3$ layer grown under BFO film as the back electrode. The field poling was performed by applying -10V DC bias to the conductive PFM probe.

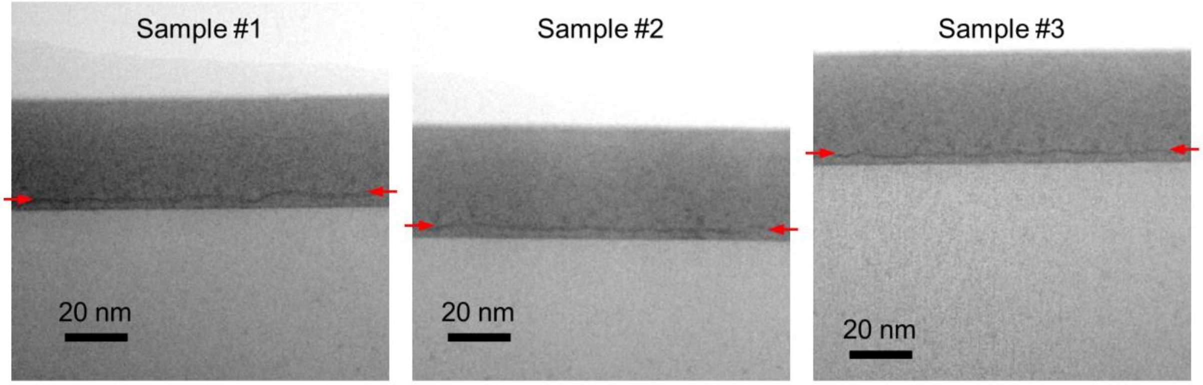

Figure S8: Reproducibility of IP-CAPBs.
